# Systemic bis-phosphinic acid derivative restores chloride transport in Cystic Fibrosis mice

**DOI:** 10.1038/s41598-022-09678-9

**Published:** 2022-04-12

**Authors:** Mélanie Faria da Cunha, Iwona Pranke, Ali Sassi, Christiane Schreiweis, Stéphanie Moriceau, Dragana Vidovic, Aurélie Hatton, Mariane Sylvia Carlon, Geordie Creste, Farouk Berhal, Guillaume Prestat, Romain Freund, Norbert Odolczyk, Jean Philippe Jais, Christine Gravier-Pelletier, Piotr Zielenkiewicz, Vincent Jullien, Alexandre Hinzpeter, Franck Oury, Aleksander Edelman, Isabelle Sermet-Gaudelus

**Affiliations:** 1 INSERM U1151, équipe 11, Paris, France; 2 Université de Paris, Paris, France; 3 INSERM U1151, équipe 8, Paris, France; 4 Molecular Virology and Gene Therapy, Department of Pharmaceutical and Pharmacological Sciences, KU Leuven, Leuven, Belgium; 5 UMR 8601, CNRS, LCBPT, Paris, France; 6 Unité de Biostatistiques, Assistance Publique Hôpitaux de Paris, Paris, France; 7 Institute of Biochemistry and Biophysics, Polish Academy of Sciences, Warsaw, Poland; 8 Laboratory of Systems Biology, Institute of Experimental Plant Biology and Biotechnology, Warsaw, Poland; 9 Laboratoire de Bactériologie-Virologie-Hygiène, Hôpital Avicenne, Bobigny, France; 10 Centre de Référence et de Compétence Maladies Rares, Mucoviscidose et maladies apparentées, Hôpital Necker Enfants malades, Paris, France; 11 European Reference Network for Rare Respiratory Diseases (ERN-LUNG), Brussels, Belgium; 12 Institut Necker Enfants Malades, 160 rue de Vaugirard, 75015 Paris, France

**Keywords:** Cell biology, Diseases

## Abstract

Mutations in the Cystic Fibrosis Transmembrane Conductance Regulator gene (*CFTR*) are responsible for Cystic Fibrosis (CF). The most common CF-causing mutation is the deletion of the 508th amino-acid of *CFTR* (F508del), leading to dysregulation of the epithelial fluid transport in the airway’s epithelium and the production of a thickened mucus favoring chronic bacterial colonization, sustained inflammation and ultimately respiratory failure. c407 is a bis-phosphinic acid derivative which corrects CFTR dysfunction in epithelial cells carrying the F508del mutation. This study aimed to investigate c407 in vivo activity in the F508del *Cftr*^*tm1Eur*^ murine model of CF. Using nasal potential difference measurement, we showed that in vivo administration of c407 by topical, short-term intraperitoneal and long-term subcutaneous route significantly increased the CFTR dependent chloride (Cl^−^) conductance in F508del *Cftr*^*tm1Eur*^ mice. This functional improvement was correlated with a relocalization of F508del-cftr to the apical membrane in nasal epithelial cells. Importantly, c407 long-term administration was well tolerated and in vitro ADME toxicologic studies did not evidence any obvious issue. Our data provide the first in vivo preclinical evidence of c407 efficacy and absence of toxicity after systemic administration for the treatment of Cystic Fibrosis.

## Introduction

The Cystic Fibrosis Transmembrane Conductance Regulator (CFTR) protein is a cAMP-dependent chloride (Cl^−^) channel encoded by the *CFTR* gene^1^. Mutations in *CFTR* are responsible for Cystic Fibrosis (CF)^2^. The most common CF-causing mutation is the deletion of the 508th amino-acid (p.Phe508del, F508del thereafter) in the first Nucleotide Binding Domain (NBD1), which compromises assembly of the different CFTR domains^3^. This results in inefficient folding of F508del-CFTR and its degradation^4^. The consequence is a dysregulation of the epithelial fluid transport in the airway epithelium, and the production of a thickened mucus favoring chronic bacterial colonization with sustained inflammation and ultimately respiratory failure^2^. Proof-of-concept studies suggest that CFTR modulators restoring the functional defects of the most frequent mutation, F508del, are associated with major clinical benefits^5^.

We have identified a bis-phosphinic acid derivative, c407 thereafter, that induces partial functional restoration of F508del-CFTR in transfected HeLa cells^7^. We recently demonstrated that this compound occupies the position of the missing p.Phe508 amino acid and stabilizes CFTR interdomain assembly. This enables its synergy with VX-809, a CFTR corrector in clinical use^8^. Therefore, c407 might be considered as a potential cotherapy of other CFTR correctors, given its mechanism of action. Moreover, this compound may also enable F508del-CFTR trafficking to the membrane by decreasing its interaction with Keratin 8^9^. Interestingly, this is associated with an anti-inflammatory effect based on inhibition of NF-κB and Wnt/β-catenin pathway^10^. This dual corrector and anti-inflammatory activity is thus interesting in the context of the CF respiratory disease that is triggered by a defective muco-ciliary clearance due to a decreased CFTR dependent chloride (Cl^−^) transport but also an overactive inflammatory response^11^.

To gain better insight into its potential clinical use, we investigated the in vivo efficacy of c407 in restoring CFTR function in the nasal epithelium of F508del mice either after short-term intraperitoneal injections, or after a long-term subcutaneous administration. Toxicological studies further completed our analysis for potential clinical use.

## Results

### c407 restores CFTR-dependent Cl^−^ transport after topical administration

**Figure 1:**
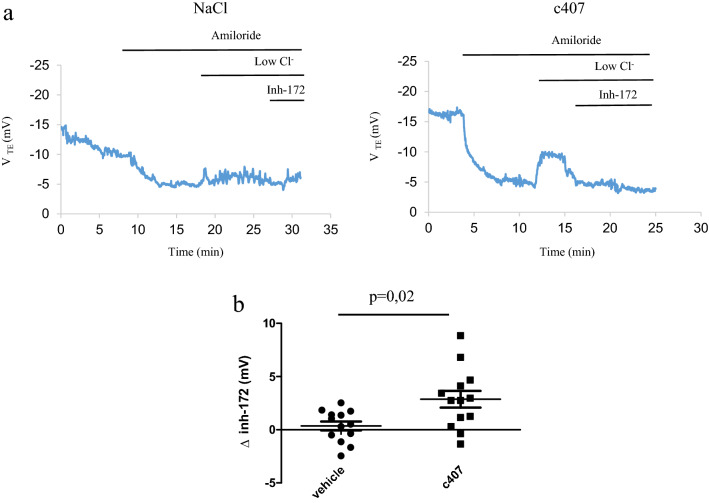
Effects of topical administration on Nasal Potential Difference in F508del *Cftr*^*tm1Eur*^ mice. (**a**) Tracing of Nasal Potential Difference performed in a F508del *Cftr*^*tm1Eur*^ mouse after topical instillation once a day for 48 h of NaCl 0.9% (left panel) or NaCl 0.9% followed by c407 10 µM (right panel). (**b**) Summary of Nasal Potential Difference changes after perfusion of Inh-172 5 µM in low Cl^−^ solution (∆ Inh-172) in mice treated with NaCl 0.9% (n = 13) or NaCl 0.9% followed by c407 10 µM (n = 13).

We first confirmed our initial result of CFTR function restoration by c407 after its topical application onto the nasal mucosa for 2 days^7^. The effect of c407 was tested at a concentration of 10 µM in F508del *Cftr*^*tm1Eur*^ mice (n = 13)^12^. Using a within-subject approach, mice underwent a series of 4 Trans-Epithelial Potential difference (V_TE_) experiments, according to our published protocol^13–15^. The Nasal Potential Difference (NPD) measurements were spaced one week apart to allow for recovery of nasal epithelia: at baseline (Day 0); at Day 7 after instillation of the NaCl vehicle; at Day 14 after instillation of c407 and at Day 21 (Supplementary Figure S1). c407 treatment significantly increased CFTR dependent Cl^−^ transport in response to perfusion of the nasal mucosa with a low Cl^−^ solution by comparison to the NaCl vehicle (p = 0.02) (Table 1, Fig. 1, Supplementary Figure S2). This response was significantly inhibited by Inh-172 (p = 0.02). After a 7-days washout, CFTR dependent Cl^−^ transport had returned to basal state (Table 1, Supplementary Figure S2). The amiloride-sensitive Na^+^ conductance (ΔAmiloride) was not statistically different between the two groups.

**Table 1:** Nasal potential difference in F508del *Cftr*^*tm1Eur*^ mice after nasal application of c407 or vehicle.

V_TE_* (mV) (mean, SEM)	Days of administration							
	D0 Baseline n = 13	D7 NaCl n = 13	D14 c407 n = 13	D21 Wash-out n = 12	p D7-D0	p D14-D7	p D21-D14	p D21-D0
Δ Amiloride	10.1 (1.1)	10.5 (1.1)	11.7 (0.9)	9.2 (0.7)	NS	NS	NS	NS
Δ Low Cl^−^	0.1 (0.4)	1.48 (0.5)	−2.1 (1)	0.7 (0.5)	NS	0.02	0.02	NS
Δ Inh-172	1.4 (0.5)	0.4 (0.4)	2.9 (0.7)	0.2 (0.05)	NS	0.02	0.01	0.02

Univariate analysis by Repeated Measures ANOVA test.

*V_TE_: Transepithelial Potential Difference.

### c407 restores CFTR-dependent Cl^−^ transport after systemic administration

In a second set of experiments, we assessed the efficacy of c407 after systemic administration. The effect was evaluated either after a short-term (72 h) intraperitoneal administration (0.124 mg/20 g, thrice a day for 3 days) to 14 F508del *Cftr*^*tm1Eur*^ and 6 KO *Cftr*^*tm1Unc*^ mice^16^, or after a long-term (22.3 mg for 28 days) subcutaneous administration to 19 F508del *Cftr*^*tm1Eur*^ mice and their 23 WT littermates.

**Figure 2:**
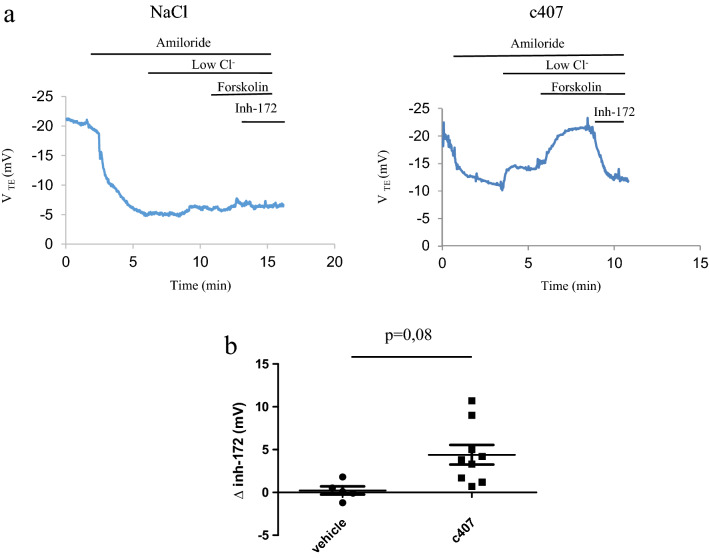
Effects of intraperitoneal administration on Nasal Potential Difference in F508del *Cftr*^*tm1Eur*^ mice. (**a**) Tracing of Nasal Potential Difference performed in a F508del *Cftr*^*tm1Eur*^ mouse after intraperitonal administration thrice a day for 3 days of NaCl 0.9% (left panel) or c407 (0.124 mg/20 g) (right panel). (**b**) Summary of Nasal Potential Difference changes after perfusion of Forskolin 10 µM followed by Inh-172 5 µM in low Cl^−^ solution (∆ Inh-172) in mice treated with NaCl 0.9% (n = 5) or c407 (n = 9).

**Figure 3:**
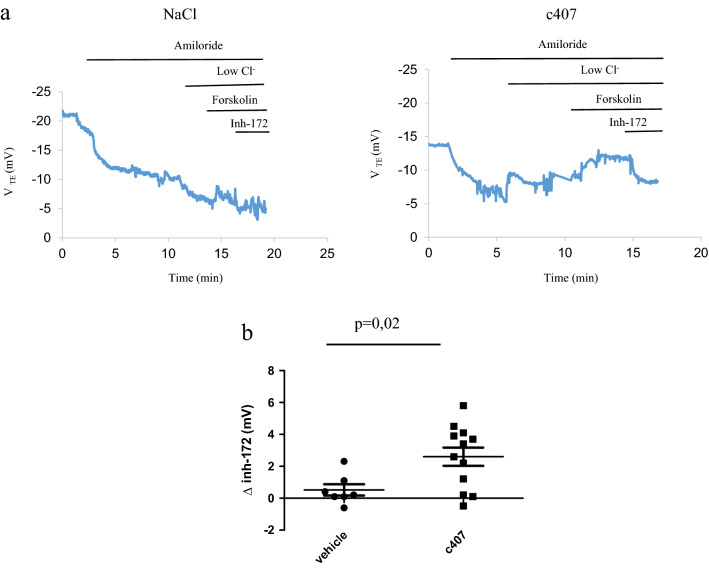
Effects of subcutaneous administration on Nasal Potential Difference in F508del *Cftr*^*tm1Eur*^ mice. (**a**) Tracing of Nasal Potential Difference performed in a F508del *Cftr*^*tm1Eur*^ mouse after subcutaneous administration for 28 days of NaCl 0.9% (left panel) or c407 (22.3 mg) (right panel). (**b**) Summary of Nasal Potential Difference changes after perfusion of Forskolin 10 µM followed by Inh-172 5 µM in low Cl^−^ solution (∆ Inh-172) in mice treated with NaCl 0.9% (n = 7) or c407 (n = 12).

The Cl^−^ transport in response to low Cl^−^ solution and Forskolin 10 µM was significantly increased in the nasal mucosa of F508del *Cftr*^*tm1Eur*^ mice after c407 intraperitoneal and subcutaneous administration (p = 0.002 and p = 0.03 respectively), as compared to mice injected with NaCl 0.9%, (Table 2). This corresponded to 93% of the WT reference level for the intraperitoneal route and 47% for the subcutaneous injection. This response was inhibited by Inh-172 and this reached a significant level for the subcutaneous route (p = 0.02) (Table 2, Fig. 2 and Fig. 3). These results were in contrast to the ones obtained in WT *Cftr*^*tm1Eur*^ and KO *Cftr*^*tm1Unc*^ mice, for which no change in CFTR activity was observed after c407 treatment (Table 2, Supplementary Figures S3 and S4). c407 treatment did not alter Na^+^ transport in any conditions.

**Table 2:** Nasal potential difference after systemic administration of c407 or its vehicle by intraperitoneal route to KO *Cftr*^*tm1Unc*^ (KO) and F508del *Cftr*^*tm1Eur*^ (F508Del) mice or subcutaneous route to F508del *Cftr*^*tm1Eur*^ and their wild type (WT) littermates.

V_TE_ * (mV) (mean, SEM)	Intraperitoneal administration						Subcutaneous administration					
	KO			F508del			WT			F508del		
	NaCl n = 3	c407 n = 3	p	NaCl n = 5	c407 n = 9	p	NaCl n = 14	c407 n = 9	p	NaCl n = 7	c407 n = 12	p
ΔAmiloride	9.4 (0.7)	11.8 (0.8)	NS	9.6 (2.5)	13.8 (1.8)	NS	3.8 (0.4)	3.6 (0.3)	NS	11.0 (0.9)	10.2 (1.3)	NS
ΔLow Cl^−^	1 (0.5)	0.4 (0.4)	NS	−0.1 (0.8)	−3.6 (0.6)	0.007	−6.3 (1)	−8.8 (1.8)	NS	0.5 (0.7)	−3.4 (1)	0.01
ΔLow Cl^−^/Forskolin	4.1 (0.6)	3.1 (0.8)	NS	−1.4 (0.8)	−7.0 (1.1)	0.002	−7.5 (1.2)	−10.8 (2.4)	NS	0.0 (0.7)	−3.5 (1.3)	0.03
ΔInh-172	−0.2 (0.2)	ND	NS	0.2 (0.5)	4.4 (1)	0.08	2.3 (0.7)	4.0 (1.2)	NS	0.5 (0.3)	2.6 (0.6)	0.02

Univariate analysis by Wilcoxon test.

CFTR functional restoration was also studied in the intestinal epithelium of F508del *Cftr*^*tm1Eur*^ mice treated with c407 by intraperitoneal administration. In c407 treated F508del *Cftr*^*tm1Eur*^ mice with (n = 9), the Short circuit Currents (IsC) induced by Forskolin/IBMx (10/100 µM) were higher than in NaCl injected mice (n = 5). However, this was not significant, due to high variability in the treated mice, as observed in NaCl *versus* c407 treated mice (ΔForskolin: 7.8(4) µA/cm^2^ *versus* 15.8(17) µA/cm^2^ (NS); ΔBumetanide: − 6(8.6) µA/cm^2^ *versus* − 10(14.8) (NS)). One third of the tissues displayed a ΔForskolin response above the highest level observed in NaCl treated mice (Supplementary Figure S5).

### Functional restoration of CFTR-dependent Cl^−^ transport by c407 is dose-dependant

Plasma concentration of c407 was determined after 28 days of subcutaneous diffusion. There was no significant difference between F508del *Cftr*^*tm1Eur*^ and WT *Cftr*^*tm1Eur*^ control mice. The target plasma concentration of 10 µM (corresponding to 3080 ng/ml), which was efficient by topical route, was not reached (Supplementary Figure S6).

The correlation between the plasma concentration of c407 and the level of Cl^−^ secretion inhibition by Inh-172 did not reach the significance level (Spearman non parametric correlation = 0.77; p = 0.08; n = 6). (Supplementary Figure S7).

### c407 restores apical expression of F508del-CFTR in F508del mice

**Figure 4:**
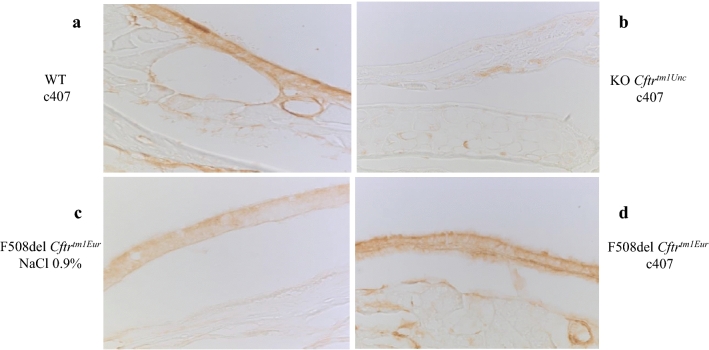
CFTR immunostaining of nasal epithelium in CF mouse models. (**a**) WT *Cftr*^*tm1Eur*^ mouse. (**b**) KO *Cftr*^*tm1Unc*^ mouse. (**c**) F508del Cftr^tm1Eur^ mouse treated with NaCl 0.9% subcutaneous administration. (**d**) F508del Cftr^tm1Eur^ mouse treated with c407, subcutaneous administration.

F508del-CFTR expression was investigated by immunohistochemistry in the nasal mucosa of subcutaneously treated mice. Figure 4 shows representative experiments from n = 3. Minimal apical staining could be observed in KO *Cftr*^*tm1Unc*^ mice in comparison to a strong staining in WT *Cftr*^*tm1Eur*^ after c407 treatment (Fig. 3a and b). Mucosa of F508del *Cftr*^*tm1Eur*^ treated by NaCl 0.9% displayed a weak predominantly cytoplasmic staining (Fig. 3c). This was in contrast to c407 treated mice which showed a marked apical staining, similar to that observed in WT mice (Fig. 3d).

### Tolerance and toxicological studies

Administration of c407 in F508del *Cftr*^*tm1Eur*^, KO *Cftr*^*tm1Unc*^ and their WT littermates was well tolerated, and no adverse effect was observed for the 3 modes of administration, based on weight gain, death rate, organ damages at autopsy, or sign of well-being. ADME-Tox studies (i.e. absorption, distribution, metabolism, excretion and toxicity) showed a good tolerance profile (Supplementary Figure S8).

The distribution coefficient (logD_7.4_, n-octanol/PBS) for c407 was very low at 0.09, which is in agreement with its high water solubility for concentrations ranging from 2 to 100 µM. The intestinal absorption was measured in apical-to-basolateral and basolateral-to-apical direction across Caco-2 monolayers and expressed by the apparent permeability coefficient (P_app_ [10^−6^ cm/s]). The coefficients were low at respectively 1.16 (± 1.42, n = 2) and 0.29 (± 0.03, n = 2), which may suggest a poor permeability after oral drug administration.

No significant inhibition effects were observed for any cytochrome P450 isoenzymes (1A2, 2B6, 2C8, 2C9, 2D6, 3A, 2C19) at 10 µM. There was no signal of genotoxicity at 5, 10, 50, and 100 µM according to AMES fluctuation test. None of the hERG, Nav1.5, and Cav1.2 channels were significantly inhibited by c407 at 10 µM (SafetyScreen87 assay) and no notable effects on hERG were observed by automatic patch clamp measurements up to 100 µM concentrations, suggesting absence of cardiac toxicity. IC_50_ values for main enzymatic targets did not show any abnormal signal.

There was no specific binding or activity modulation by c407 at 10 µM concentration, for specific pharmacological targets, i.e. GPCR receptors, transporters, ion channels, nuclear receptors, kinases, and other enzymes suggesting limited adverse drug reactions. Only monoamine oxidase type B, (MAO-B, P27338, http://www.uniprot.org/uniprot/P27338) was inhibited by 50%.

## Discussion

We provide in vivo evidence that the c407 CFTR corrector induces a functional restoration of CFTR in the nasal epithelium of F508del mice after systemic short and long term administration.

We previously reported c407’s corrector efficiency in different cell lines including HEK, BHK and HeLa cells^7,8^. This was based on Western blots for CFTR expression and two functional assays, patch clamp, and Short circuit Current, which provided evidence for CFTR activity restoration. The effect of c407 was confirmed, at a lesser extent, in primary respiratory cultures of nasal and bronchial origin^7,8^. We recently performed molecular docking and dynamics simulations combined with site-directed mutagenesis to investigate c407’s mechanism of action^8^. We showed that c407 improves CFTR folding by stabilizing F508del-Nucleotide Binding Domain (NBD1) during the co-translational folding and occupying the position of the missing p.Phe508 side chain after whole domains assembly thus reinforcing the flawed Intra Cellular Loop ICL4-NBD1 interface. Remarkably, this refolding effect synergizes with VX-809, the corrector used in Orkambi® dual therapy. Whereas, per se*,* c407, and its G1 derivative, have little CFTR corrector activity in human respiratory cells, they could be used in combination to other CFTR correctors to stabilize the NBD1-ICL4 interface of misfolded CFTR.

c407 corrector effect might be also additive to other, less understood effects. It has been shown that F508del-NBD1 binds to intermediary filaments and more specifically to Keratin 8 (K8) and that diminishing the expression of K8 or disrupting K8 interaction with F508del-CFTR increases F508del-CFTR function^9^. c407 decreases the interaction between F508del-CFTR and Keratin 8^7^ suggesting a scaffolding effect of the latter, whose reduction in turn, might facilitate the processing of misfolded CFTR to the plasma membrane^9,17^. Altogether, c407 might act as a protein–protein interaction inhibitor and this might be useful in other misfolding mutations than F508Del.

We already published the effect of c407 by topical nasal application in F508del mice^7^. We did not replicate the protocol used in this paper, which compared two small groups of mice (4 treated *versus* 3 non treated). In the protocol used in the present study, each mouse was its own control. NPD was measured in a larger number of mice (n = 13) every 7 days: at basal state, after NaCl 0.9% application, c407 application, and after 1 week of wash out. Our main aim was to study whether c407 corrects the CFTR-dependent activity at the organism level. To do this, we used systemic administration to avoid the first pass effect that is inherent to oral administration.

By using NPD to investigate CFTR activity longitudinally in vivo, we confirm the bioactivity of c407. When administrated by intraperitoneal and subcutaneous route, c407 induced an increase in CFTR-dependent Cl^−^ transport reaching 93% of WT levels for acute intraperitoneal administration and 47% for long term subcutaneous administration. The significant change in the response to Inh-172, together with the absence of functional effect in WT and KO mice provide strong evidence that this modification was due to changes in CFTR activity. This was correlated with an increased localization of F508del-CFTR at the apical surface of nasal epithelial cells compared to control treated animals. At this point however, we cannot discern which cells within the nasal mucosa correlated with the functional rescue measured by NPD. In light of the recent identification of ionocyte as the major source of CFTR expression and function in murine lungs and evidence of the role of secretory cells in human epithelia, this warrants further investigation regarding the contribution of each airway cell type in the functional recovery of CFTR by c407^18,19^.

Additional proof of concept for c407 activity is its dose-dependent relationship as assessed by the relation between c407 concentration and CFTR dependent Cl^−^ transport. This is also suggested by the fact that intraperitoneal short-term administration at a high dosage (0.124 mg/20 g, thrice a day for 3 days) allowed to reach a higher correction than subcutaneous long-term administration at a lower dosage (22.3 mg for 28 days). Interestingly, we report here for the first time the efficiency of a long-term, 1-month subcutaneous administration of a CFTR corrector in a CF murine model.

Very few studies that explore the effect of F508del correctors have been performed in the murine CF model. Lubamba et al. showed the role of PDE5 inhibitors in restoring chloride transport function of F508del-CFTR after intraperitoneal or inhaled administration^20,21^. Veit et al. explored combinations of different CFTR correctors and confirmed these observations in CF F508del mice^22^. Our data add further evidence that measurement of electrophysiological changes in the nasal mucosa of the CF mouse can be valuable in investigating long-term CFTR correction at the organism level. Albeit in vitro experiments provide evidence for the effect of the molecule and eventually its mechanism of action, the activity of the drug after its metabolization in a complex living organism remains unknown, including activity of its metabolites. CF mice, whose nasal mucosa share similar electrophysiological properties with respect to human CF mucosa, allow to expand the effect of CFTR correctors to that of a whole organism.

In agreement with absence of toxicity in mice, toxicology data showed that the compound should be well tolerated after systemic administration. First, c407 did not inhibit any CYP isoenzymes and therefore is not anticipated to induce serious side effects via a metabolism-based drug-drug interaction mechanism. Second, no important toxicity signals were evidenced either in bacterial mutation assays, which estimate the mutagenic potential, or by blocking cardiac ion-channels such as potassium hERG (human-ether-a-go-go), sodium (Nav1.5) or L-type calcium (Cav1.2) ion-channels. Interestingly, only a few biological off-targets were seen in the i*n vitro* pharmacological profiling assay. IC_50_ values for main enzymatic targets did not show any significant signal apart for monoamine oxidase type B. This may be a collateral effect and should only prove toxic if the drug passes across the blood–brain barrier, which must be evaluated.

The anti-inflammatory effect of c407 was shown in F508del-Cftr osteoblasts where F508del-*Cftr* mutation decreases osteoblast differentiation and function as a consequence of increased NF-κB and decreased Wnt-β-catenin signaling^23^. Treatment of murine F508del-Cftr osteoblasts with c407 increased the expression of Wnt responsive genes and corrected the exacerbated NF-κB transcriptional activity and this was associated with rescued osteoblast activity and bone formation after in vivo short-term treatment in F508del-Cftr mice^10^. Altogether, this dual CFTR correction and anti-inflammatory activity might be interesting in the context of respiratory diseases which combine defects in CFTR and increased NF-kB mediated inflammation. This is the case for CF as well as other respiratory diseases such as Chronic Obstructive Pulmonary Disease, secondary consequence of smoking or alpha1 antitrypsin deficiency^24^.

The limitation of this study is the discrepancy between the result obtained in the murine animal model and in the F508del homozygous human primary respiratory cells, where c407 activity was inconsistent. This human-mouse differential effect might have several explanations. First, the mouse F508del-cftr is partially correctly refolded thus facilitating the effect of correctors, in contrast to human F508del-CFTR^25^. Second the CFTR expressing cells in mice are mainly ionocytes^18^ and non-ciliated, microvillus-rich brush cells^26,27^. This is in contrast to humans where secretory cells are the main CFTR expressing cells followed by basal cells^19^. These differences at the protein and the cellular level may explain why a CFTR modulator might have a different impact in the mouse and humans. Finally, the efficacy of oral administration of c407 in CF mice will need to be verified as this route is the preferential one in therapeutic applications. Considering the low permeability coefficient of c407 in the Caco-2 assay, if oral administration of c407 in CF mice would turn out not to be effective, this would create a severe hurdle for its systemic administration. Inhaled topical administration might thus be considered. .

## Conclusion

Altogether, this preclinical study confirms the bioactivity of the c407 F508Del-CFTR corrector, with a good efficiency and long-term tolerance. The absence of toxicity and the pleiomorphic effect of c407 suggest that this compound might be clinically relevant and implementable not only for CF but also for other respiratory diseases associated with the loss of function of misfolded CFTR.

## Methods

### Mouse strains

We used congenic mice homozygous for the F508del mutation, F508del *Cftr*^*tm1Eur*^, and their wild-type (WT) littermates from the FVB/N background^12^, CFTR knock-out mice KO *Cftr*^*tm1Unc*^ and their wild-type littermates from the B6;129 background^16^. All were obtained from CDTA (Cryopreservation, Distribution, Typage et Archivage animal, Orléans, France), and housed at Animal Care Facility of Necker Hospital, Paris. Mice were fed a fiber-free diet and their drinking water contained a laxative (Colopeg 17.14 g/l; Bayer Santé Familiale, France) to avoid intestinal obstruction. Experiments were performed according to European Community regulations for the use of animals in research and the study was approved by the Ministry of Research (PROJET MESR No. 01345.03). Authors complied with ARRIVE guidelines. Mice were anesthetized by intraperitoneal injection of ketamine (133 mg/kg; IMALGENE 1000, MERIAL, France) and xylazine (13.3 mg/kg; Rompun 2%, BayerPharma, France) before in vivo experimentations. Survival, prevalence of ileus, growth and general aspect were recorded to assess compound toxicity.

### Nasal potential difference measurements

Nasal Potential Difference (NPD) measurements were performed as previously described with minor modifications^13–15^. Briefly, the Trans-Epithelial Potential difference (V_TE_) was assessed between a reference Ag/Ag electrode connected to a subcutaneous needle with an agar bridge, and an exploring Ag/AgCl electrode connected to the nasal mucosa through a double-lumen polyethylene catheter (0.5 mm in diameter). One lumen of the catheter was filled with HEPES-Krebs-buffer (140 mM NaCl, 6 mM KCl, 10 mM HEPES, 10 mM glucose, 1 mM MgCl_2_, 2 mM CaCl_2_, pH adjusted to 7.4). The following solutions were perfused through the second lumen (1) HEPES-Krebs-buffer (Ringer) for basal V_TE_ measurement, (2) 100 µM amiloride in Ringer solution (Sigma-Aldrich, USA) to block ENaC Sodium (Na^+^) absorption (Δ Amiloride); (3) a low Cl^−^ solution (where 140 mM NaCl was replaced by 140 mM sodium gluconate) to drive Cl^−^ secretion (Δ Low Cl^−^); (4) a low Cl^−^ solution supplemented with forskolin 10 µM (Sigma-Aldrich, USA) to activate Adenyl cyclase and induce production of cAMP which will activate CFTR (Δ Forskolin), Δ Low Cl^−^/Δ Forskolin representing the sum of Δ Low Cl^−^ and Δ Forskoline; and (5) a low Cl^−^ solution with forskolin and Inh-172 5 µM to inhibit CFTR activation (Δ Inh-172). V_TE_ was recorded using an averaging analog to digital converter supplied by Logan Research Ltd, Rochester, Kent, UK. Forskolin was used only in the second set of experiments concerning systemic administrations. The change in nasal V_TE_ after Inh-172 was considered as the index of CFTR-activity as it specifically shows the proportion of Cl^−^ secretion which is due to CFTR.

NPD measurements were performed at baseline and after topical administration by nasal instillation of 50 µL of c407 10 µM once a day for 2 days, or after systemic administration either by intraperitoneal route (0.124 mg/20 g), thrice a day for 3 days (short-term administration), or subcutaneous route by continuous administration for 28 days (33 µg/h, reaching the maximal concentration of 22.3 mg in the 200 µl pump volume) (long-term administration) (Supplementary Figure S1). For this latter mode of administration, we used an osmotic pump (Alzet micro-osmotic pump, Model 1002), surgically installed subcutaneously in the back of 8 week-old mice for 28 days. The vehicle NaCl 0.9% was administered with identical volume for the control group. The target was a serum concentration of 10 µM, which was efficient by topical administration.

### Immunostaining of nose sections

The experiments were adapted from Vidović et al.^14^. Briefly, entire heads were fixed in 4% paraformaldehyde, decalcified with 450 mM EDTA (pH = 8), for 4 days and cryopreserved in 15% followed by 30% sucrose solution. The nose was cut into 3 pieces, embedded in Neg50 (Thermo Fisher Scientific, Saint Louis, USA) and frozen in liquid nitrogen. Cryosections of 6 μm were first incubated with 3% H_2_O_2_ to block endogenous peroxidase activity. After blocking in 10% goat serum, sections were incubated overnight at 4 °C with a polyclonal rabbit CFTR antibody (MPCT-1), raised against the 23 C-terminal amino acids of CFTR (kindly provided by Robert Dormer, University of Wales, Cardiff, UK) at a dilution of 1:100. After incubation with biotin-labeled secondary antibody and streptavidin-HRP, detection was performed using 3,3-Diaminobenzidine (DAB) substrate. Sections were mounted in Dibutylphthalate Polystyrene Xylene (DPX) medium (Sigma, Saint Louis, Missouri, USA) without counterstaining to allow better visualization of positively stained regions marked by a brown precipitate. Nasal sections from KO *Cftr*^*tm1Unc*^ mice were used as a negative control. CFTR positive cells were visualized using a Leica Biopoint 2 light microscope. Brightness and contrast were optimized using Adobe Photoshop CS6.

### Short circuit current of intestinal epithelium

After anesthesia, mice were killed by exsanguination via cardiac puncture. A segment of colon from proximal (caecum) to distal part (rectum) was excised, rinsed with 0.9% NaCl, cut in four pieces and opened longitudinally to be mounted in Ussing Chamber, so that a surface area of 0.02 mm^2^ was exposed. The tissue was bathed on each side by 2.5 mL of (CaCl_2_ 0.19, KCl 0.35, MgCl_2_ 0.2, NaCl 7.48, NaH_2_PO_4_ 0.06, Na_2_HPO_4_ 0.05, Hepes 2.38, D-Glucose 1.8, NaHCO_3_ 1.7, g/L) maintained at 37 °C and gassed with 95% O_2_–5% CO_2_. Passive ion transport across the epithelium was abolished by clamping the potential to zero using a WPI DVC-1000 voltage clamp (World Precision Instruments, Stevenage, UK) and Ag-AgClelectrodes. The Short-circuit current (Isc) was measured with EVC4000 Precision V/I Clamp (World Precision Instruments) and registered using Power Lab 4/30 workstation (AD Instruments, Castle Hill, Australia). Transepithelial resistance (RT) was measured by applying a 15 mV pulse. During continuous recording of Isc (in voltage-clamp mode) following inhibitors and activators were added in the Ussing chamber at the apical or basal side after stabilization of baseline Isc: amiloride 100 µM to the apical side (Sigma-Aldrich, USA) to block Na^+^ absorption; the cocktail of forskolin/IBMx 10 µM/100 µM (Sigma-Aldrich, USA) on both side to specifically activate CFTR and bumetanide 100 µM (Sigma-Aldrich, USA) to block Cl^−^ gradient and decrease CFTR activity.

### c407 serum concentration determinations

c407 was quantified by an UPLC-MS/MS system, an Ultimate 3000 UPLC and a TSQ Vantage MS/MS detector (Thermo Fischer, Waltham, Masssachusetts, USA). Briefly, after adding 50 µl of the deuterated isotope (100 ng/ml) to 100 µL of plasma, 500 µL of acetonitrile was added for protein precipitation. Samples were then vortex-mixed for 30 s, and subsequently centrifuged at 20,000*g* for 10 min. The supernatant was then evaporated to dryness under a stream of nitrogen and the dry residue was reconstituted with 200 µl of mobile phase (ammonium acetate 10 mM: methanol; 80:20). Ten µL were then injected to the UPLC-MS/MS system. Chromatographic separation was performed with a Kinetex C-18, 100 × 2.1 mm column (Phenomenex, Le Pecq, France) and the mobile phase flow was set at 0.4 mL/min. The monitored m/z transitions were 311 to 169 for c407 and 315 to 172 for its deuterated isotope. Spray voltage, vaporizer temperature and capillary temperature were 4000 V, 350 °C and 300 °C respectively. The calibration range was 1–10 000 ng/mL.

### ADME-Tox tests

Absorption, Distribution, Metabolism and Excretion and toxicity ADME-Tox assays were performed by Eurofins Scientific at Discovery Services, St Charles, USA; CEREP, Celle-Lévescault, France (http://www.eurofins.com and http://www.cerep.fr). The experiments were performed in accordance with Eurofins validation Standard Operating Procedure.

### Statistical analysis

Continuous variables are presented as mean (Standard Deviation (SD)). Between-group comparisons were evaluated for qualitative variables by Chi-square test or Fisher exact test if required, and for quantitative variables by Student’s t test, after normal distribution was checked by Shapiro–Wilk test, or non-parametric Wilcoxon signed-rank test in case of no normal distribution. We used a Repeated Measures ANOVA test in cases of repeated measurements in the same animal. For each test, the null hypothesis was rejected for a p-value < 0.05. Data management and statistical analysis were performed using R Statistical software (version 3.2.0; R Foundation for Statistical Computing, Vienna, Austria).

## Supplementary Information

Supplementary Figure 1.

Supplementary Figure 2.

Supplementary Figure 3.

Supplementary Figure 4.

Supplementary Figure 5.

Supplementary Figure 6.

Supplementary Figure 7.

Supplementary Figure 8.

Supplementary legends.
