# Supplementary material for: Systemic bis-phosphinic acid derivative restores chloride transport in Cystic Fibrosis mice
Source: Sci Rep. 2022 Apr 12;12:6132. doi: 10.1038/s41598-022-09678-9 (PMC9005718; doi:10.1038/s41598-022-09678-9)
Supplement: Supplementary file 1 — Supplementary Figure 1. [file 41598_2022_9678_MOESM1_ESM.ppt]

## Slide 1
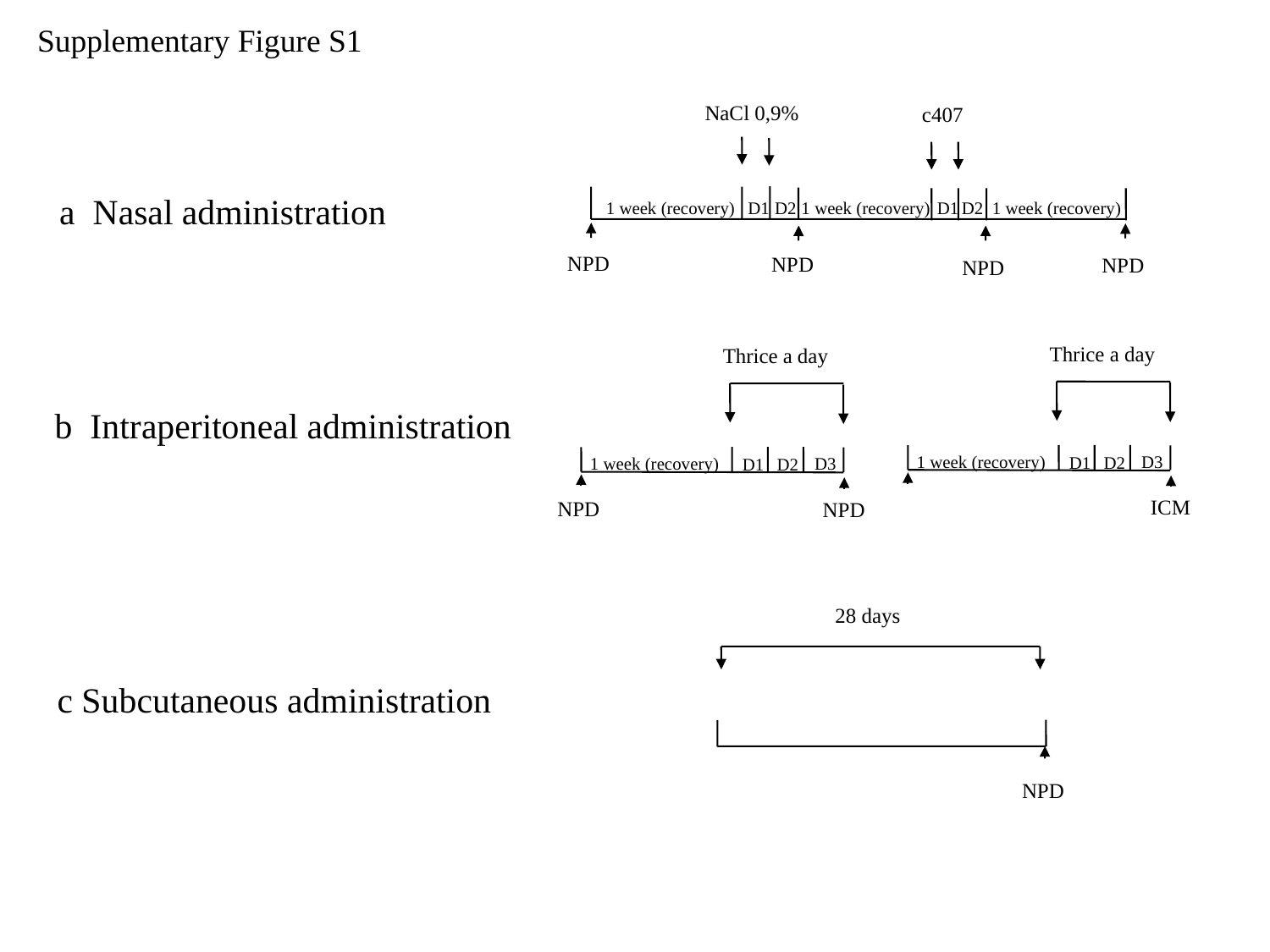

Supplementary Figure S1
 NaCl 0,9%
c407
1 week (recovery)
D1
D2
NPD
NPD
D1
D2
1 week (recovery)
1 week (recovery)
NPD
NPD
a Nasal administration
Thrice a day
1 week (recovery)
D3
D1
D2
ICM
Thrice a day
1 week (recovery)
D3
D1
D2
NPD
NPD
b Intraperitoneal administration
28 days
NPD
c
c Subcutaneous administration
