# Supplementary figures and images for: Systemic bis-phosphinic acid derivative restores chloride transport in Cystic Fibrosis mice
Source: Sci Rep. 2022 Apr 12;12:6132. doi: 10.1038/s41598-022-09678-9 (PMC9005718; doi:10.1038/s41598-022-09678-9)

## Slide 1
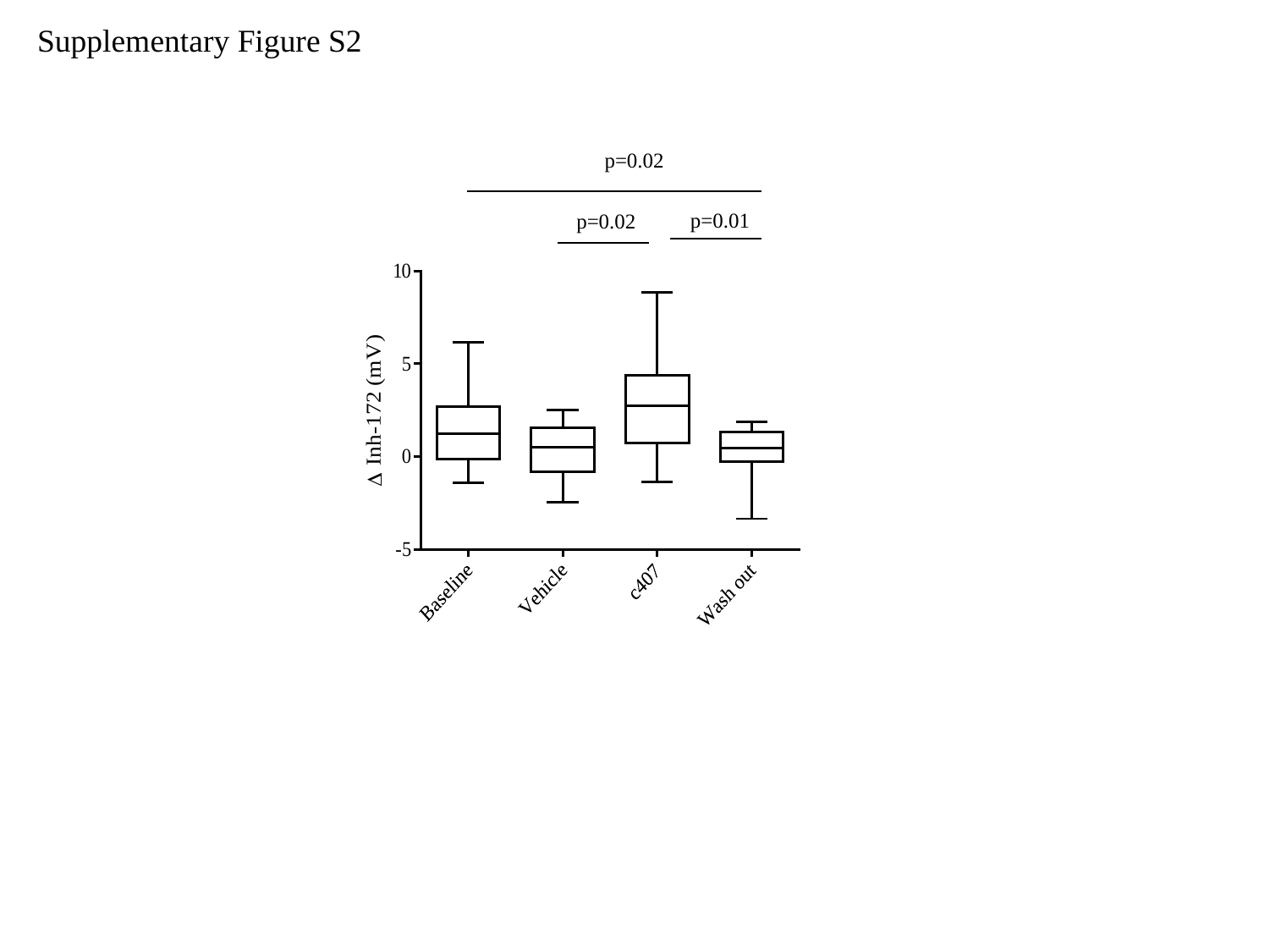

Supplementary Figure S2
p=0.02
p=0.01
p=0.02

Supplement: Supplementary file 2 — Supplementary Figure 2. [file 41598_2022_9678_MOESM2_ESM.pptx]

## Slide 1
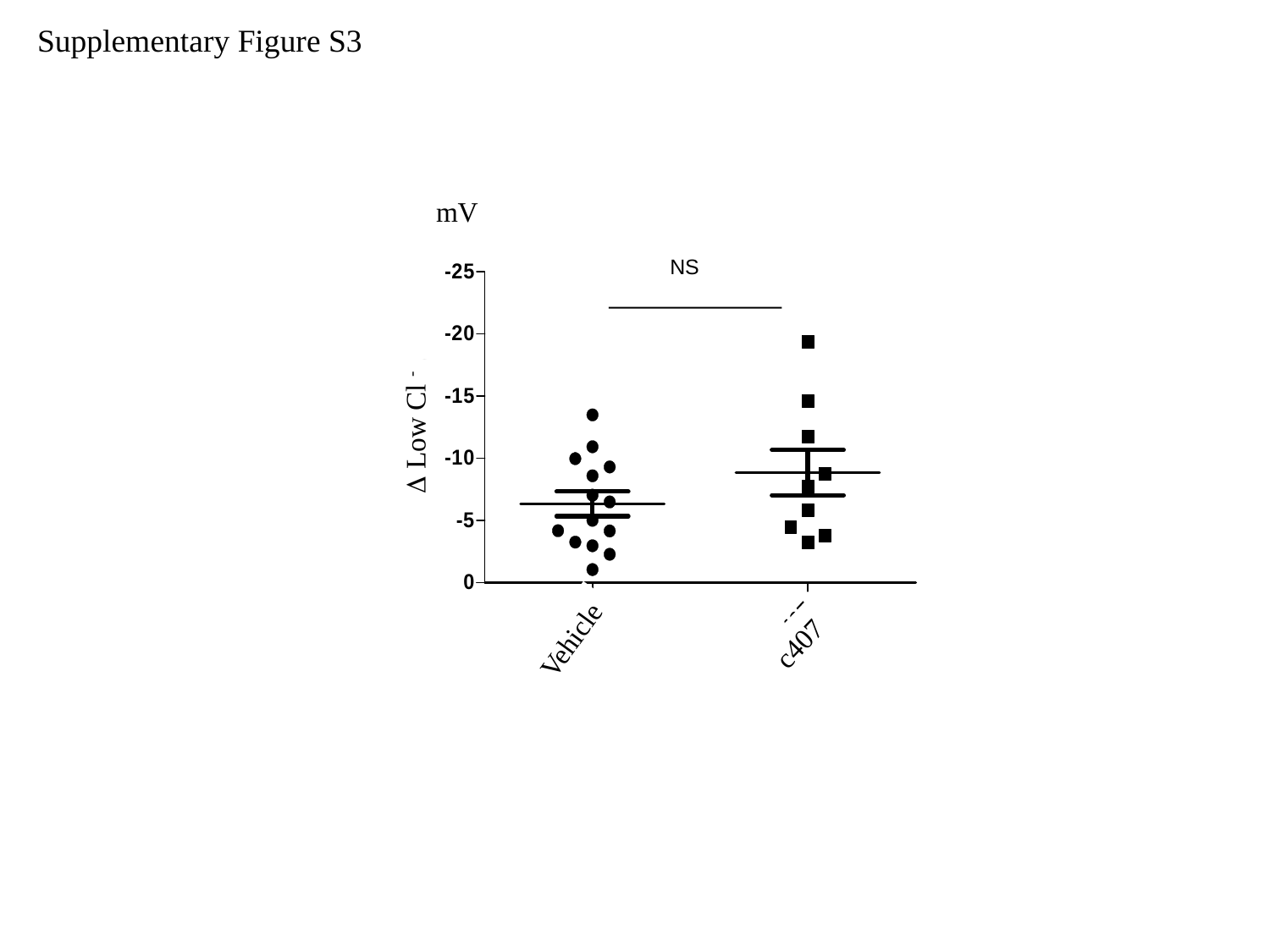

Supplementary Figure S3
mV
NS
Δ Low Cl -
Vehicle
c407

Supplement: Supplementary file 3 — Supplementary Figure 3. [file 41598_2022_9678_MOESM3_ESM.ppt]

## Slide 1
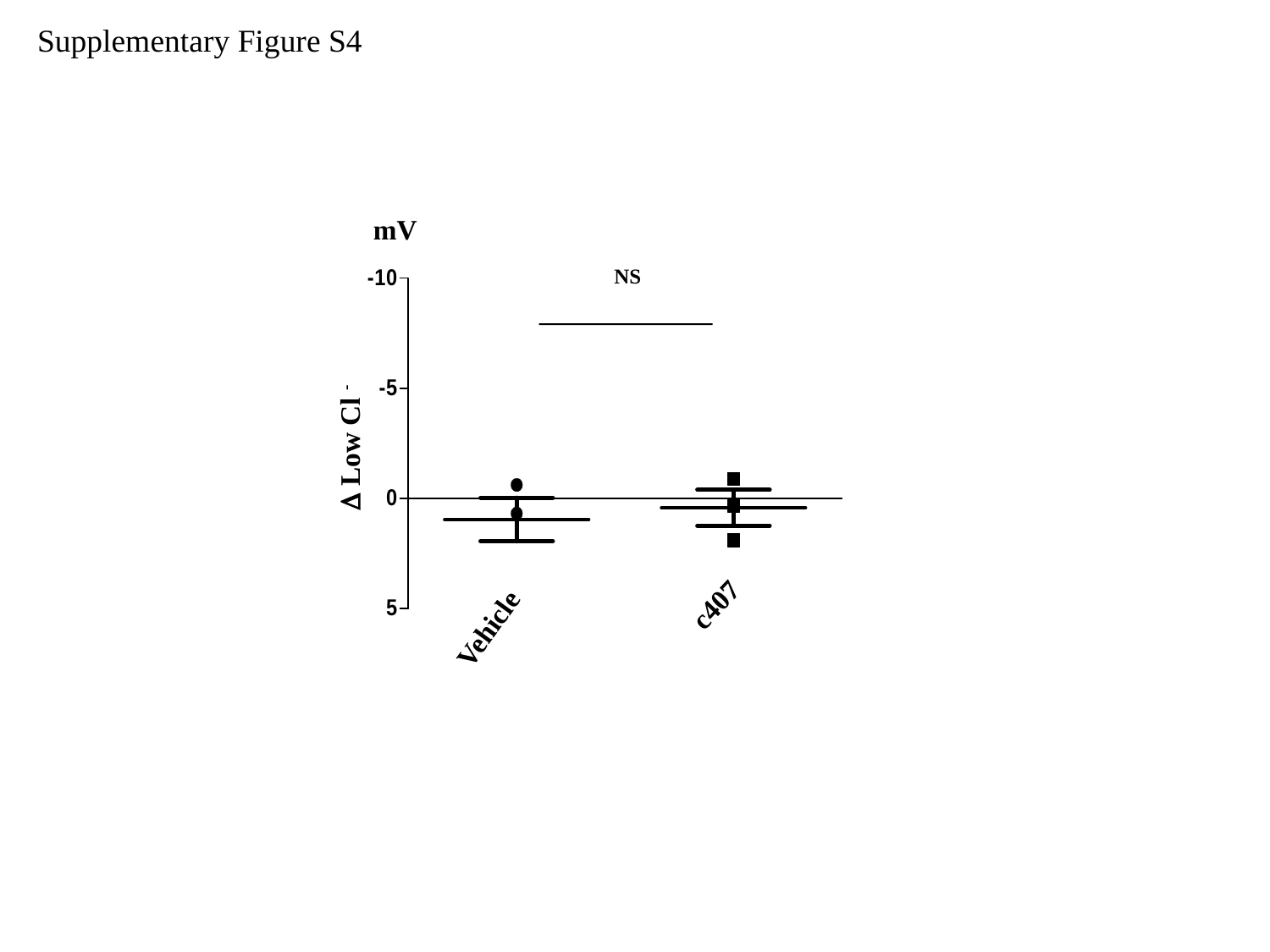

Supplementary Figure S4
mV
NS
Δ Low Cl -
c407
Vehicle

Supplement: Supplementary file 4 — Supplementary Figure 4. [file 41598_2022_9678_MOESM4_ESM.ppt]

## Slide 1
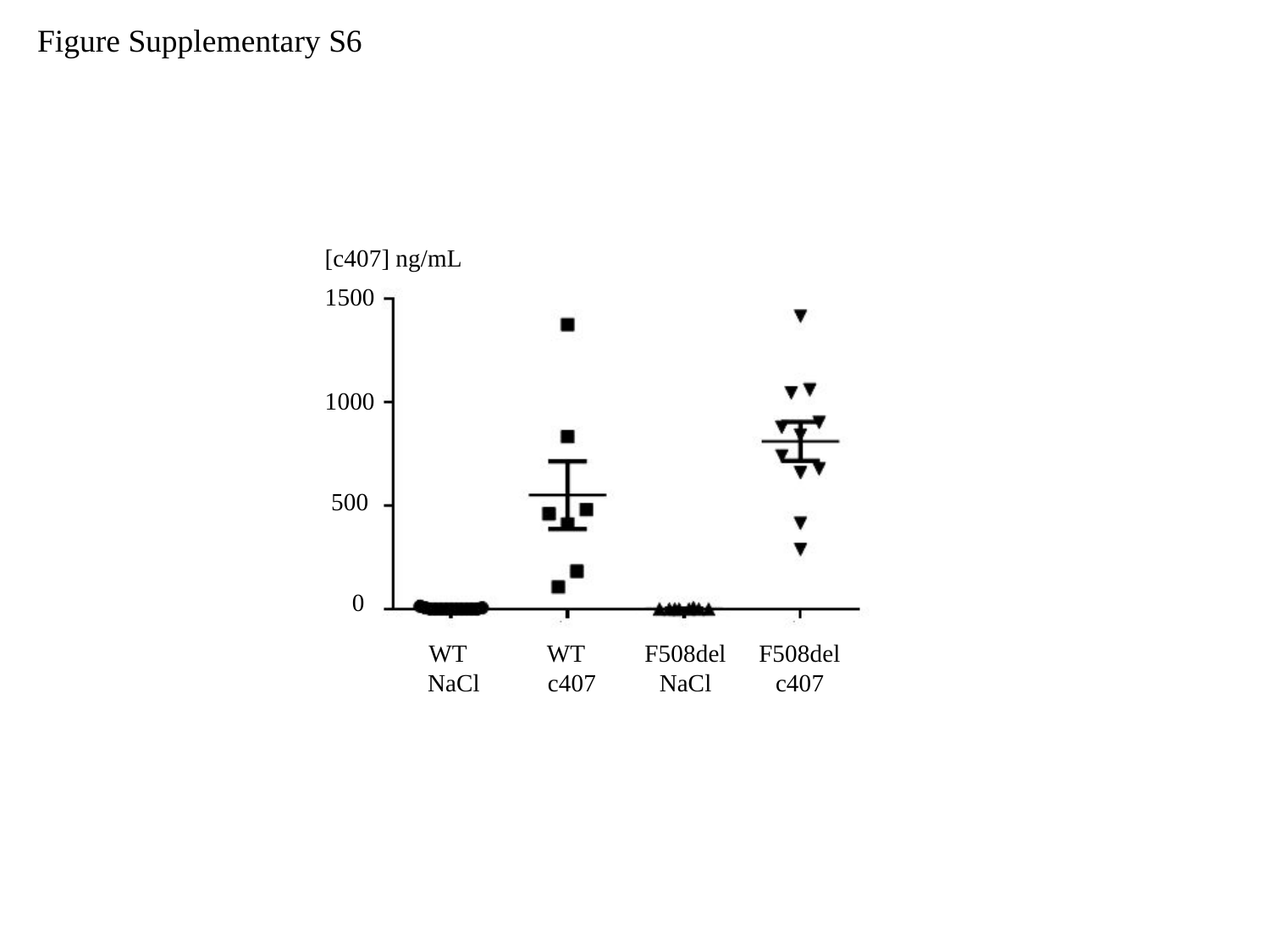

Figure Supplementary S6
[c407] ng/mL
1500
1000
500
0
WT
 NaCl
WT
 c407
F508del
NaCl
F508del
c407

Supplement: Supplementary file 6 — Supplementary Figure 6. [file 41598_2022_9678_MOESM6_ESM.ppt]

## Slide 1
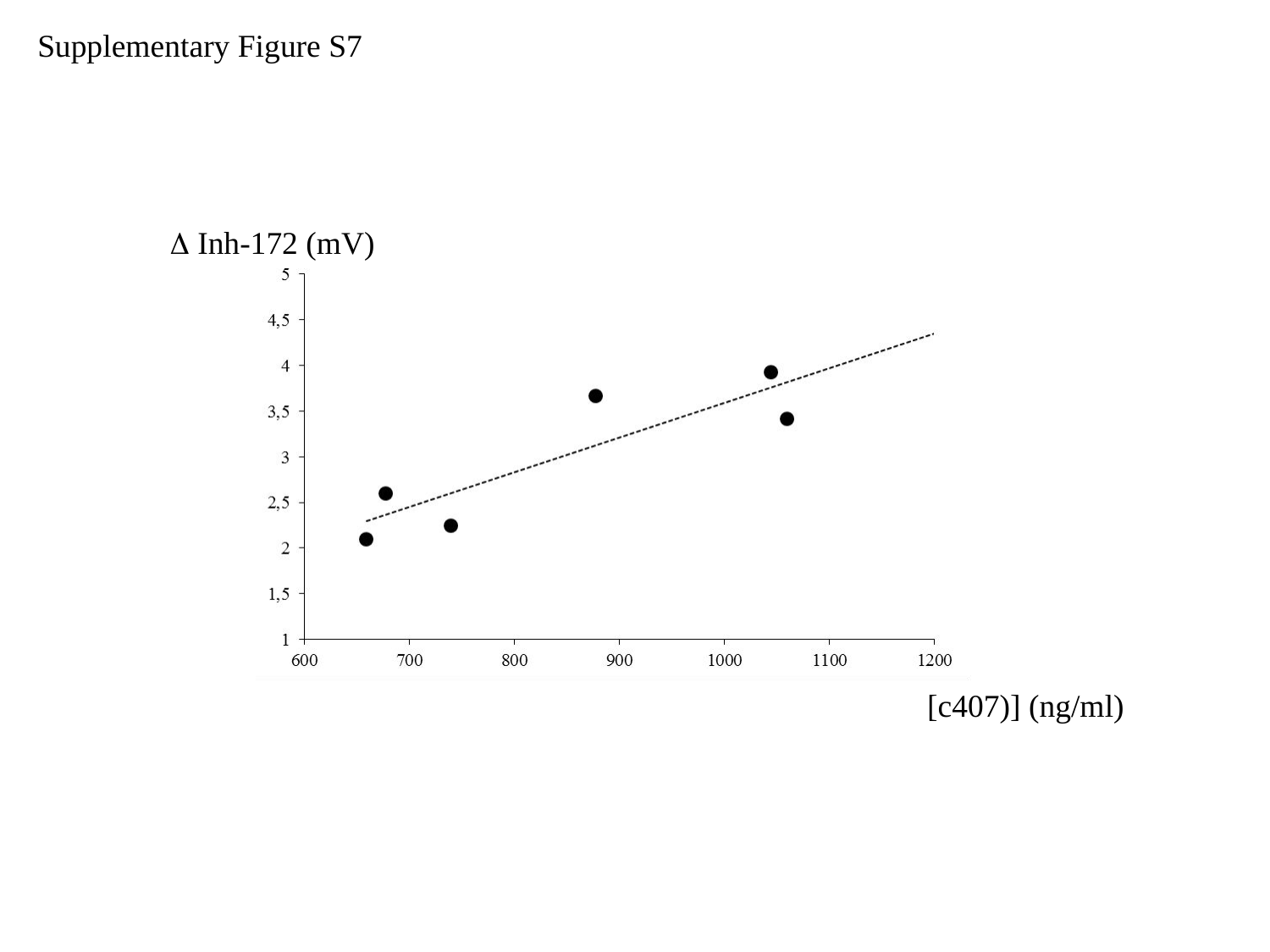

Supplementary Figure S7
Δ Inh-172 (mV)
[c407)] (ng/ml)

Supplement: Supplementary file 7 — Supplementary Figure 7. [file 41598_2022_9678_MOESM7_ESM.ppt]

## Slide 1
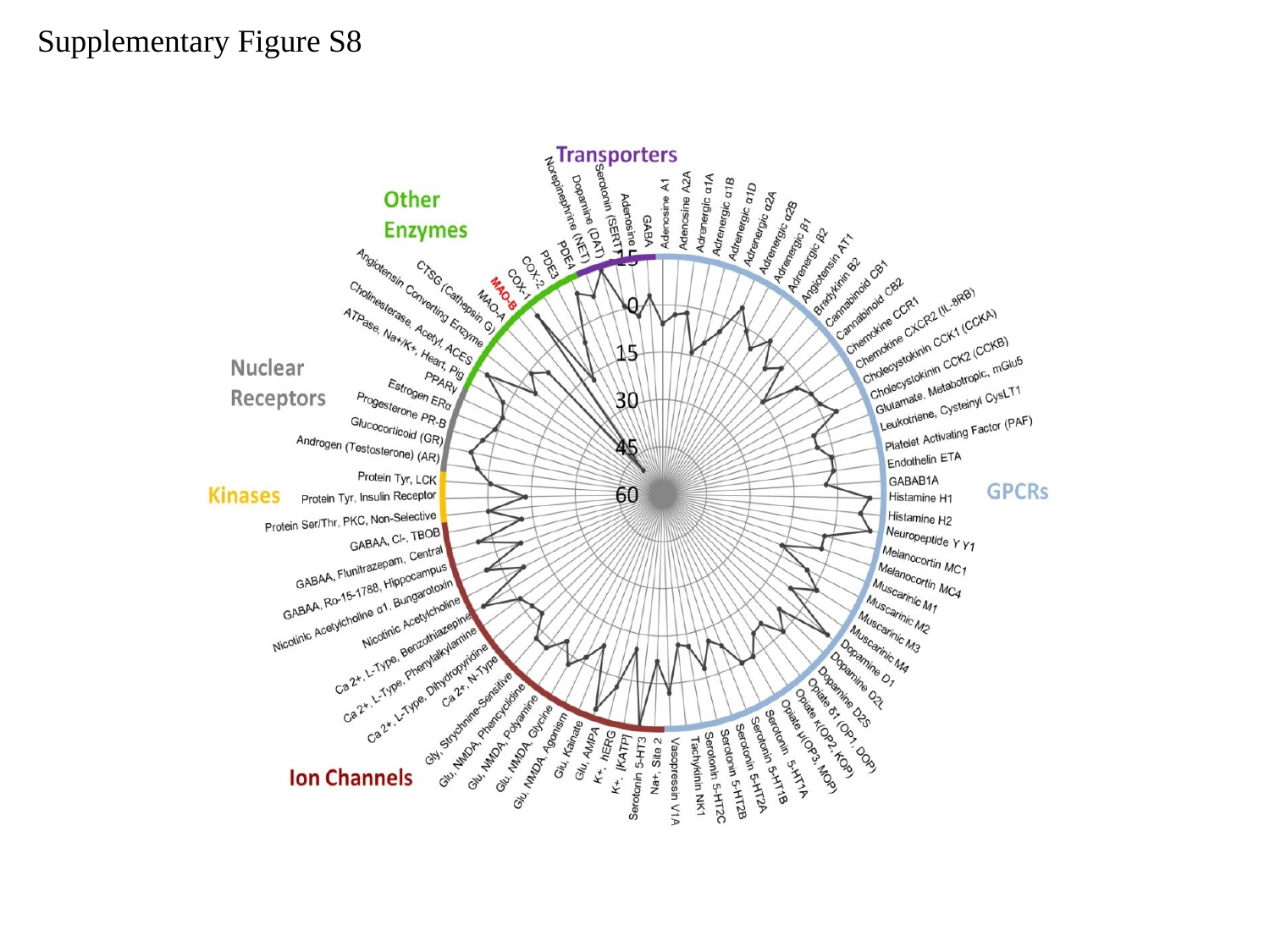

Supplementary Figure S8

Supplement: Supplementary file 8 — Supplementary Figure 8. [file 41598_2022_9678_MOESM8_ESM.ppt]
