# Supplementary material for: Systemic bis-phosphinic acid derivative restores chloride transport in Cystic Fibrosis mice
Source: Sci Rep. 2022 Apr 12;12:6132. doi: 10.1038/s41598-022-09678-9 (PMC9005718; doi:10.1038/s41598-022-09678-9)
Supplement: Supplementary file 5 — Supplementary Figure 5. [file 41598_2022_9678_MOESM5_ESM.ppt]

## Slide 1
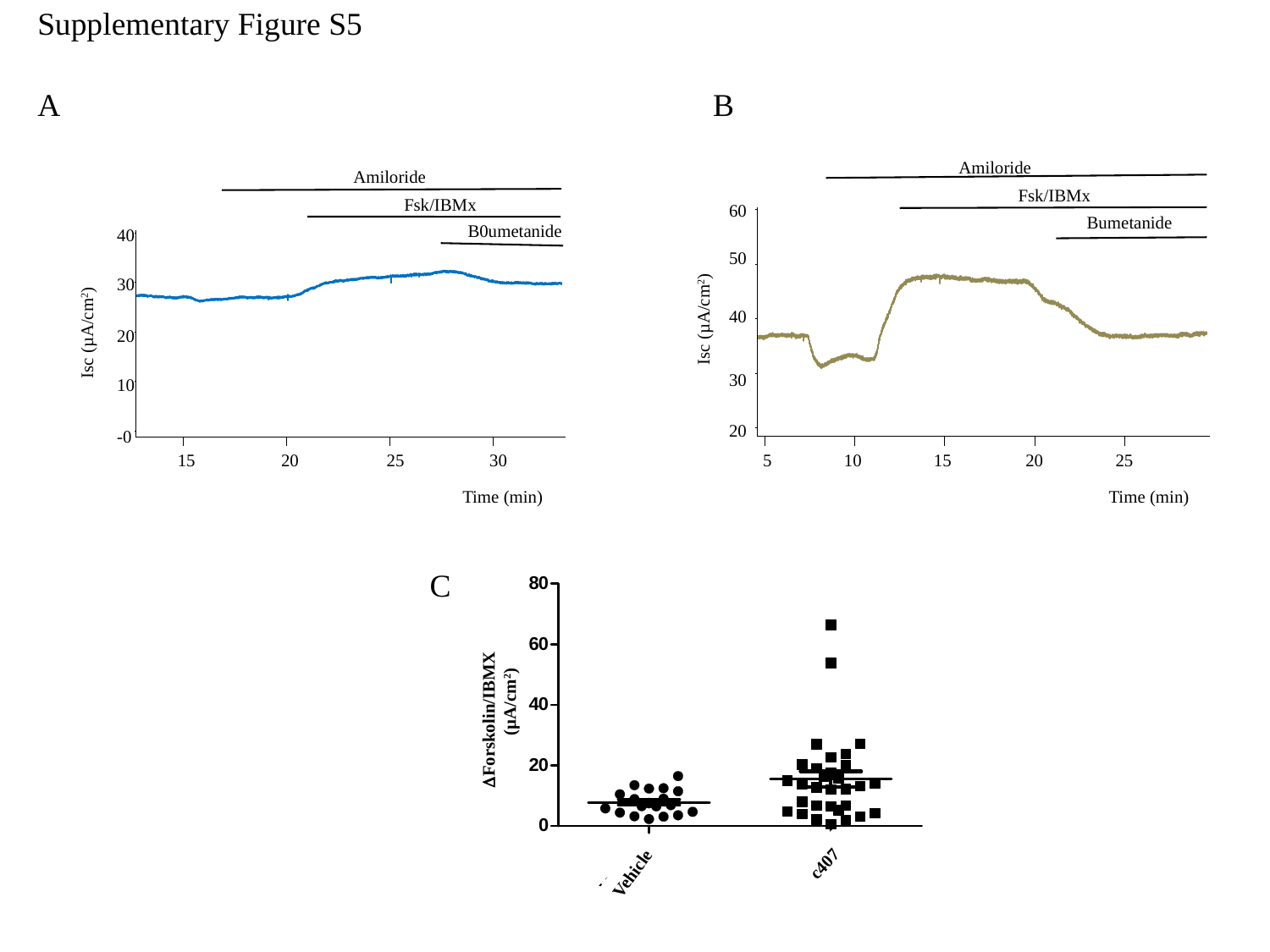

Supplementary Figure S5
B
A
Amiloride
Amiloride
Fsk/IBMx
B0umetanide
40
30
20
10
-0
15
20
25
30
Isc (µA/cm2)
Time (min)
Time (min)
ΔForskolin/IBMX (µA/cm2)
Fsk/IBMx
60
50
40
30
20
5
10
15
20
25
Bumetanide
Isc (µA/cm2)
C
c407
Vehicle
