# Supplementary material for: Systemic bis-phosphinic acid derivative restores chloride transport in Cystic Fibrosis mice
Source: Sci Rep. 2022 Apr 12;12:6132. doi: 10.1038/s41598-022-09678-9 (PMC9005718; doi:10.1038/s41598-022-09678-9)
Supplement: Supplementary file 9 — Supplementary legends. [file 41598_2022_9678_MOESM9_ESM.docx]

**Systemic bis‑phosphinic acid derivative restores chloride transport in Cystic Fibrosis mice**

Mélanie Faria da Cunha, Iwona Pranke, Ali Sassi, Christiane Schreiweiss, Stéphanie Moriceau, Dragana Vidovic, Aurélie Hatton Mariane Carlon, Geordie Creste, Farouk Berhal, Guillaume Prestat, Romain Freund, Norbert Odolczyk, Jean Philippe Jais, Christine Gravier-Pelletier, Piotr Zielenkiewicz, Vincent Jullien, Alexandre Hinzpeter, Franck Oury, Aleksander Edelman^,^Isabelle Sermet-Gaudelus

**Supplementary Figure S1. Overview of the different protocols of c407 administration in the murine Cystic Fibrosis model**

**a.** Topical application. c407 10µM or its vehicle NaCl 0.9% was administered by nasal instillation of 50 µl once a day for 2 days to F508del Cftr*^tm1Eur^* mice. Nasal Potential Difference (NPD) was measured every 7 days at basal state, after NaCl 0.9%, c407 application, and treatment discontinuation.

**b.** Intraperitoneal injection. c407 or its vehicle NaCl 0.9% was administered thrice a day for 3 days (0.124 mg/20g) to F508del Cftr*^tm1Eur^* mice, KO *Cftr^tm1Unc^* mice**.** NPD was measured at basal state and after treatment. Intestinal Current Measurement (ICM) was performed at the end of the 3-day treatment in a second group of F508del Cftr*^tm1Eur^* mice treated with c407 or Nacl 0.9%.

**c.** Subcutaneous diffusion. c407 or its vehicle NaCl 0.9% was administered continuously to F508del Cftr^tm1Eur^ mice and their WT littermates (22.3 mg for 28 days). NPD was measured at basal state, and after 28 days of administration.

**Supplementary Figure S2. c407 topical application significantly increases CFTR activity in the nasal mucosa of**  **F508del Cftr*^tm1Eur^* mice.**

Summary of the NPD results obtained every 7 days in 13 F508del Cftr*^tm1Eur^* mice at baseline, after topical application once a day for 2 days of 50 µl of NaCl 0.9%, c407 10µM, and after 7 days wash out. The change in Nasal Potential Difference (NPD) after perfusion of the mucosa with the specific CFTR inhibitor Inh-172 is shown. Univariate analysis by repeated measures ANOVA test.

**Supplementary Figure S3. c407 has no effect on CFTR activity in WT CFTR mice.**

Summary of the NPD results obtained, in WT *Cftr^tm1Eur^* mice after subcutaneous injection of the vehicle NaCl 0.9% (n=14) or c407 (22.3 mg for 28 days) (n=9).

**Supplementary Figure S4. c407 has no effect on CFTR activvity in KO CFTR mice**

Summary of the NPD results obtained in KO *Cftr^tm1Unc^* mice after intraperitoneal administration thrice a day for 3 days of the vehicle NaCl 0.9% (n=3) or c407 (0.124 mg/20g) (n=3).

**Supplementary Figure S5.** **Short circuit current (IsC) experiments in colon of F508del *Cftr^tm1Eur^* mice treated with c407 by intraperitoneal administration.**

A and B) Representative IsC tracing of distal colon sampled from a F508del *Cftr^tm1Eur^* mouse

treated by intraperitoneal administration thrice a day for 3 days of the vehicle (NaCl 0.9%) (left panel) or c407 (0.124 mg/20g) (right panel).

C) Plots of the IsC modification after addition of forskolin/IBMx (10µM/100µM) in Ussing chamber of distal colon sampled from F508del *Cftr^tm1Eur^* mice treated by intraperitoneal administration thrice a day for 3 days of vehicle (NaCl 0.9%)(n=5) or c407 (0.124 mg) (n=9). Mean number of 3 tissues studied per mouse.

**Supplementary Figure S6. c407 seric concentration**

Seric concentration after 28 days continuous subcutaneous injection of NaCl 0.9% or 22.3 mg of c407 in WT and F508del *Cftr^tm1Eur^* mice.

**Supplementary Figure S7. Correlation between serum concentration of c407 and CFTR**

**activity in F508del *Cftr^tm1Eur^* mice treated by subcutaneous administration of c407.**

Correlation between c407 seric concentration and NPD change after perfusion of the nasal mucosa by a low chloride solution containing Forskolin 10 µM and CFTR_inh_172 5µM). (Spearman coefficient:0.771; p=0.08; n=6)**.**

**Supplementary Figure S8. In vitro toxicity evaluation of c407.**

The c407 corrector was screened against 87 human proteins (anti-targets), such as GPCR receptors, transporters, ion channels, nuclear receptors, kinases, and other enzymes to evaluate potential undesirable effect of drug reaction (Eurofins Panlabs, Inc.). The biochemical assay results are presented on “a radar chart” as the percent inhibition of specific binding or activity for specific targets at 10 μM concentration of c407.
